# A Case of Banti's Disease, with Autopsy

**Published:** 1903-03

**Authors:** J. Michell Clarke

**Affiliations:** Professor of Pathology, University College, Bristol; Physician to the Bristol General Hospital.


### A CASE OF BANTI'S DISEASE, WITH AUTOPSY.

BY

J. MICHELL CLARKE, M.A., M.D. (Cantab.), F.R.C.P.,

*Professor of Pathology, University College, Bristol;*

*Physician to the Bristol General Hospital.*

---

THE case is as follows:—

Laura B., æt 19, a servant. She had always lived in the country, and came of a healthy family. She had never had any bad illness. Four years previously she noticed an increase in the size of her abdomen, and on being examined this was found to be due to an enlarged spleen. Since then she had suffered from weakness and anæmia; once she had a very severe attack of epistaxis, which lasted for two hours. She had always been a teetotaller.

Twelve days before admission she vomited nearly half a pailful of dark blood, and eleven hours later a further quantity, not so much as the first. She had no pain, and has never suffered from pain in the stomach or bowels, nor from dyspepsia or vomiting. Two days before the attack the catamenia, which had been absent for eight months, came on in a normal way. Occasionally, about once in two or three months, she had had profuse sweats at night. Except for symptoms referable to anæmia, *e.g.* shortness of breath, palpitation, pallor of face, and slight œdema of ankles, she made no other complaint.

On admission, February 24th, 1902, she was white and thin. The skin showed some sparsely-scattered, greyish pigment spots. Pulse, 134; small, regular, and weak. Hill and Barnard's sphygmometer indicated 120 on the scale. The breath had a peculiar, faint ethereal odour. Blood, pale; red cells, 1,171,000; white, 7,473. Hæmoglobin, 12 per cent. The red corpuscles were extremely pale, of all shapes and sizes, with some poikilocytes. Chief point in stained specimen was a moderate increase in large and small lymphocytes.

On examination, lungs healthy. Heart's apex was in sixth space, half-inch outside nipple line. A wavy impulse extended from second to sixth space. The left ventricle was dilated. The first sound at the apex was modified, and accompanied by a systolic murmur, audible at the lower angle of left scapula. Some ascitic fluid present in abdomen. The lower edge of the liver was not felt, the liver dulness reaching only to a finger's breadth above lower border of ribs. Stomach area of resonance obliterated. The spleen was enormously enlarged, firm and hard, not tender, smooth, and its notch plainly felt. It reached from the seventh rib above to close upon the iliac crest below, and forwards an inch to the right of the umbilicus. A soft souffle was audible over the spleen, but no friction sound. The urine was pale, 1,015, and contained a slight trace of albumin; no sugar; no casts; a few cystals of oxalate of lime. No retinal hemorrhages. Treatment by rest in bed, and the administration of iron, arsenic, strychnine, and digitalis.

On March 4th. Blood examination: hæmoglobin, 12 per cent.; red corpuscles, 1,200,000, varying very much in shape and size; a very few nucleated red cells (normoblasts).

On March 12th. Heart dulness extended outwards nearly to anterior axillary line, and nearly two inches to right of sternum. Murmurs as before. Liver dulness began above at fifth rib, and its lower edge could be plainly felt below the lower border of the ribs. The spleen had increased in size, extending downwards to about two inches above the pubes. There was marked œdema of the legs, and the ascites had increased. The skin had a clear waxy tint. In a stained specimen of blood, coarsely granular eosinophil cells numbered 4 per cent. of leucocytes; no myelocytes were seen, and only one or two nucleated red cells.

March 19th. Blood hæmoglobin, 14 per cent.; red cells, 1,320,000; white, 5,250 to cm.; differential count: polymorphonuclear cells, 58 per cent.; eosinophils, 3 per cent.; mononuclear lymphocytes, 39 per cent., of which 11 per cent. were large hyaline lymphocytes; 1 coarsely granular basophil cell; 1 myelocyte. No nucleated red cells seen in this specimen.

March 26th. Hæmoglobin, 20 per cent.; red cells, 1,600,000; white, 5,200.

April 4th. Over eight pints of blood-stained fluid removed from abdomen: it contained blood corpuscles, but did not coagulate. The heart sounds had a galloping rhythm. The spleen had still further enlarged, reaching the pubes below and extending to the right to line half way between umbilicus and anterior superior spine of ilium.

April 14th. Blood had a peculiar bright pink colour, and was very pale. Hæmoglobin, 22 per cent.; red cells, 1,900,000; white, 3,500. Differential count showed polymorphonuclear cells, 56 per cent.; small lymphocytes, transitional cells, and large lymphocytes, 43.5 per cent., of which about 18 per cent. were large lymphocytes, one myelocyte, and one eosinophil cell to 400. Red corpuscles varied in size; few or no poikilocytes, some stained darkly, degenerated. One nucleated red was seen. The puncture with a needle bled very profusely. Optic discs and retinæ very pale (anæmia), but not otherwise abnormal. The urine, besides containing a trace of albumin, showed a marked urobilin band. No bile was present in it.

On April 26th eleven pints of fluid were removed from the abdomen. The patient had improved, and the blood state had also improved, as the red cells were now 2,100,000 to cm., and the hæmoglobin had gradually increased to 28 per cent. Leucocytes, 5,000. Differential count showed same proportions. No myelocytes. The œdema had disappeared from the legs, and the spleen was slightly reduced in size; it still had the same characters, being hard, firm, smooth, and not tender. The heart murmurs were about the same, the dilatation less. The heart dulness did not extend over the right border of sternum, and only one-and-a-half inches to left of nipple line, whilst the pulsation over the cardiac area had diminished. The liver reached to the lower border of the ribs. The patient slept well, was comfortable and free from pain when propped up in bed, and had a good appetite. She was now taking tr. digitalis, ♏ xv., and liq. arsenicalis, ♏ viii. (the dose of the latter having been gradually increased) with iron, *ter in die*.

On May 2nd she had an attack of severe diarrhœa, which passed off, and the temperature rose to 100°. On May 4th and

5th the temperature rose to 105°. There were no rigors, and no lesion could be found in any part of the body to account for the fever. The temperature fell rapidly to normal on the 6th in the same unaccountable way. On May 10th the patient vomited five pints of blood, and died a few hours afterwards.

It should be added that throughout the illness, except as stated on May 2nd to 6th, the temperature was irregular, varying between normal and 100° F.; there was never any jaundice, nor any reaction for bile in the urine.

Owing to difficulty in getting leave, the *post-mortem* examination was not made until fifty-six hours after death. Body emaciated; profoundly anæmic. Each pleura contained ½ pint, the pericardium 4 oz., and the peritoneum 4 to 5 pints clear yellowish serum, which had not the peculiar bright colour seen in the exudations in pernicious anæmia, and did not give the urobilin band. The lungs were pale, rather rusty in colour, but not indurated; there was some hypostatic congestion; trachea and bronchi healthy. Heart, 13 ozs. Both ventricles were dilated, the right also hypertrophied. Valve segments, normal. Heart-muscle, pale; some excess of subpericardial fat, especially infiltrating wall of right ventricle. No fatty degeneration of heart wall or papillary muscles. Liver weighed 3 lbs. 7 ozs., smooth on surface; on section, a pale bright yellow colour, almost primrose, with a number of slightly raised rounded nodules, varying in size from a millet seed to a pea; these varied in number in different places: consistence slightly tougher than normal. The spleen was enormous, weighed 3 lbs. 13 ozs.; it was smooth, dark in colour, and the notch well marked. On section, it was a pale brick-red, smooth, not unduly hard; fibrous structure not obviously increased; the Malpighian bodies deficient in number. It contained one very large pale infarct. The capsule of the spleen was slightly thickened; a chain of enlarged glands, dark in colour, the size of a large bean or larger, extended from the hilum of the spleen along the upper border of the pancreas to the middle line. The pancreas was large, and rather hard; white in colour. The supra-renals were bright yellow, and their medulla almost diffluent (? *post-mortem* change). The kidneys—right, 5 ozs.; left, 6 ozs.;—were pale and anæmic on section, the capsules stripped well, leaving a smooth pale-yellow surface with injected stellate veins, otherwise they looked normal. No thymus was found. Thyroid large, on section reddish, and contained glistening material, somewhat resembling the organ in Graves's disease. Pharynx normal. The œsophagus at and just above the cardiac end was richly beset with dilated and varicose veins. The stomach was full of blood-clot: the hemorrhage had taken place from a small eroded surface amongst the large varicose veins at the cardiac orifice. No sign of ulcer, past or present, was found. The upper part of the intestine also contained a quantity of blood-clot. The intestines, large and small, appeared healthy. The

2

uterus and ovaries were normal. The marrow of the tibia appeared abnormally dark red in colour and free from fat; that of the ribs was healthy.

*Microscopical Examination.*—Pieces of the various organs were hardened in Müller's fluid, and subsequently in alcohol, and sections stained with hæmatoxylin, with hæmatoxylin and orange G. and rubin, with hæmatoxylin and eosine, by Van Gieson's method, and with osmic acid. Lungs, heart, pancreas, supra-renals, thyroid, and ovaries showed no abnormal changes. Spleen: The trabeculæ and connective tissue of the organ showed a general increase. The vessels everywhere, especially those running with the trabeculæ, and also the capillary vessels of the sinuses, showed chronic congestion. In places there were considerable amounts of blood pigment, not contained in cells. The small vessels of the pulp showed pretty generally proliferation of the lining endothelium; there was also an endo-capillaritis of some capillaries, the lumen of which was stuffed with cells. In some parts in the sinuses there were considerable numbers of large cells, with clear protoplasm and large round or oval nuclei not staining so deeply as small lymphocytes, these were apparently the large lymphocytes (pale hyaline mononuclears) of blood: it was doubtful whether these cells, which are normally present in the spleen, were in excess. There were no giant-cells, nor evidence of proliferation of endothelium of sinuses. The Malpighian bodies were very few in number; some were stuffed with lymphocytes, others contained much fewer, and possibly there was some excess of fibrous tissue in these latter. The vessels of the Malpighian bodies and trabeculæ were less affected than those of the pulp, but the endothelium in some appeared swollen. In gland from hilum of spleen the trabeculæ and framework of gland were thickened, the vessels injected, and there had been hemorrhage into some of the sinuses. In some sinuses there were many large cells with deeply-staining nuclei, a few of still larger size, which looked like large lymphocytes, contained more than one nucleus. Liver: In the portal canals there was a slight excess of a fine fibrillar connective tissue, with a moderate number of round cells. This was not universal, but more marked in certain parts; and where most marked it was encroaching on the liver lobules, cutting off peripheral liver cells, which in some cases were elongated, and in a few had just divided. The liver cells were cloudy, their nuclei stained normally. The central veins of the lobules were a little dilated, with some excess of connective tissue around them. Kidney: The only changes in this organ were that the glomerular capillaries were injected, and in some glomeruli the epithelium of the capsule and that overlying the tuft showed slight proliferation of cells, with a little exudation into the capsule, and slight thickening of it. These changes were slight only. There was also, in some parts only, desquamation and proliferation to moderate extent of epithelium of first part of convoluted tubes. There was no reaction for fat with osmic acid in liver, kidney, or heart; and no iron reaction in liver, spleen, or kidney.

The only remark to be made in addition to the above description is that the changes in the spleen are slighter in character than one would expect from its enormous size, and that the Malpighian bodies might be regarded as *relatively* few, owing to enormous increase in the pulp. The slight kidney changes correspond to those found in excretion of a not very intense poison; and, taking into consideration the enlarged glands in hilum of spleen and the changes in the liver, they might be regarded as secondary to passage of a toxin.

This case corresponds, then, with Banti's disease in its essential features, which, according to Banti, are, clinically, profound and progressive anæmia with enlargement of the spleen, and secondary cirrhosis of the liver with ascites. There are frequent hemorrhages, especially gastro-intestinal and nasal.[1] According to Murrell, however,[2] Banti speaks only of a notable tumefaction of the spleen and also of the liver, and describes the following as essential features: (1) A progressive idiopathic anæmia; (2) Grave disturbance of all the organic functions; (3) Irregular fever; (4) Hemorrhage; and (5) a fatal termination. Briefly speaking, certain cases of splenic anæmia are attended in the late stages with ascites, with or without cirrhosis of the liver, and sometimes jaundice.

The special diagnostic features of splenic anæmia, it will be remembered, are: the presence of an enlarged spleen, often of enormous size, with anæmia rather of the chlorotic type, without any increase, but in many cases a decrease, of the white corpuscles, and tendency to repeated and large hemorrhages. The disease runs a very prolonged course, and occasionally may cease to advance, or be arrested for long periods of time. In most cases it is, however, progressive to a fatal termination, which may occur from hemorrhage, or from gradual exhaustion; and the termination may be attended with cirrhosis of the liver, giving rise to ascites: the latter is the particular variety of splenic anæmia generally known as Banti's disease.

Dr. Dreschfeld[3] considers that splenic anæmia includes a number of affections which have two symptoms in common,

[1] Barr, *Lancet*, 1902, ii. 493. [2] *Lancet*, 1902, i. 1177.

[3] *Brit. M. J.*, 1902, i. 719.

namely, anæmia and an enlarged spleen; that the splenic anæmia of adults, in which there are besides usually severe and repeated hemorrhages, is often only the early stage of Banti's disease, marked enlargement of the liver appearing at a later stage; though in several cases of splenic anæmia, which were evidently cases of Banti's disease that died in the early stage, the liver showed *post-mortem* distinct signs of cirrhosis. Professor Osler, in a recent paper before the Association of American Physicians,[1] said that some of his cases of splenic anæmia had lasted ten to twelve years. The blood changes had been those of a chloranæmia, with, in six or eight cases, an exceedingly low leucocyte count. In ten cases hemorrhages from the stomach had been a marked and characteristic feature. Pigmentation of the skin had occurred in seven cases. In the late stages there had been a change in the size of the liver—sometimes an increase, sometimes a diminution. Jaundice might be an early symptom, and was not necessarily associated with cirrhosis of the liver. Recurrent attacks of anæmia, with jaundice and ascites, had marked a recent case. In only one out of three spleens examined had the changes supposed to be characteristic of the disease been present.

Next, as to the blood in the cases described as splenic anæmia, the conditions found present considerable variations as regards number of red corpuscles. In some of Osler's cases above mentioned the number was as high as 4,000,000, and the hæmoglobin 30 per cent. In many other cases the number has been as low as 1,000,000, and in one case (Cabot) 384,000: possibly the differences may be partly accounted for by the low blood-counts having been made towards the fatal termination of the disease, and the high ones in non-progressive stages of it. Generally speaking, there seems to be a diminution of the red corpuscles, but in most cases not an excessive one, and a relatively low value of corpuscles in hæmoglobin; *i.e.* an anæmia of the type found in chlorosis. As to the white corpuscles, they seem to be never increased; and in many cases there is a leucopenia, with a relative increase of lymphocytes to polymorphonuclear cells: this was the condition in my case, and the

[1] *Med. Rec.*, 1902, lxi. 750.

later counts showed striking increase in the number of large lymphocytes or pale hyaline mononuclear cells.

With regard to gastro-intestinal hemorrhages, since they occur in cases in which there is no cirrhosis of the liver, they cannot be attributed entirely to congestion produced by this cause. In my case, however, the fatal hemorrhage is undoubtedly to be connected with the varicose veins at the œsophageal orifice; and in one other case on record death was due to bleeding from an ulcerated varicose vein in this situation. The frequent occurrence of epistaxis, and the observation noted above that the prick of a needle in my patient bled extremely freely, point to the alteration in the blood as an important factor in the occurrence of hemorrhage. It has also been suggested by Osler that the hæmatemesis is mechanical, and due to venous engorgement of those areas of the gastric mucous membrane which are drained by the vasa brevia and gastro-epiploica sinistra veins, which again open into splenic vein passing through gastro-splenic omentum.[1]

Ascites has been present in some cases without cirrhosis of the liver. In the above case, the cirrhosis of the liver was in an early stage, and would seem insufficient to produce such a large amount of ascites, but one must remember that here there are other factors at work. There is the condition of the blood, with its influence upon the nutrition of the capillary walls, probably favouring transudation of fluid; and there is the dilated and weak heart, causing stagnation in the venous circulation. These conditions, each of which alone might not be adequate to produce ascites, in co-operation might easily do so. In other cases, towards the end of the disease, the same factors would be at work, and the occurrence of ascites may be thus accounted for, even when the degree of cirrhosis is small. Possibly in my case there was a further factor, in that the enlarged glands spreading from the hilum of the spleen may have compressed a branch or branches of the portal vein, and so aided in the production of ascites.

With regard to the cause of the cirrhosis of the liver in this disease, Banti[2] divides primary enlargements of the spleen

[1] *See* Rolleston, *Clin. J.*, 1902, xix. 407.
[2] *Riforma med.*, March 1st—5th, 1901; *Brit. M. J.*, 1901, i. Epitome, p. 93.

into the spleno-medullary leukæmic type, in which "the spleen alters in structure, manufactures leucocytes of unusual quality, and pours them into the circulation, radically changing the leucocytic ratio of the blood, besides elaborating certain soluble toxins." "In the other three types of splenomegaly the leucocytes are not increased. The altered function shows itself chiefly in the elaboration of certain toxic products which are poured into the circulation and act powerfully on the red corpuscles. In splenomegaly with jaundice a true hæmolytic action takes place; in splenic anæmia and in splenomegaly with hepatic cirrhosis . . . the splenic toxins, in their passage across the liver, sometimes induce fatty degeneration, sometimes cirrhosis of that organ, sometimes a mixed process is set up."

Chauffard [1] also insists on the hepatic cirrhosis being secondary to poisons formed in the spleen, and regards the portal vein as being formed of two branches, the intestinal and the splenic, either of which may convey poisons to the liver capable of inducing cirrhosis.

With regard to the nature of splenic anæmia, this is at present very obscure. The resemblances of the changes found in the spleen, in some cases, to those met with in the glands in lymphadenoma (Hodgkin's disease), as described by some authors, have led them to regard splenic anæmia as a lymphadenoma of the spleen; and it may be recalled that similar changes are met with in the glands related to the spleen.

There are difficulties, however, in regarding the disease as a primary one of the spleen. Professor Osler, indeed, says that the only point in favour of the view that the spleen might have something to do with this disease was that a certain number of cases had recovered after removal of the organ. Dr. James Barr's view is, that Banti's disease is due to a vaso-motor paresis of the splanchnic area, either in whole or in part, and that this paresis arises from disease of the visceral sympathetic ganglia. As a consequence, there is great engorgement of the abdominal viscera, especially of the spleen and liver; increased hæmolysis, with consequent oligochromæmia and oligocythæmia. The increased blood-supply to these organs eventually leads to

[1] Quoted by Rolleston, *loc. cit.*

fibrosis and lessened function. In support of this are the changes found by Banti in the semilunar ganglia and solar plexus, and also in the ganglia of the neck; *i.e.* lymphoid infiltration of ganglia, with degeneration of ganglion cells and nerve fibres leading from them.

There is still so much uncertainty about the exact position of Banti's disease in relation to the various forms of secondary anæmia and of splenic enlargement, and so much to be learnt of the etiology of these anæmias and of the causation of enlarged spleens, that at present it is impossible to come to any satisfactory conclusion as to the real nature of the disease and its position in nosology.

In conclusion, I may add that the absence, in such severe and long-continued anæmia as was present in the above case, of any fatty degeneration is worthy of remark, and forms a contrast, on the one hand to chlorosis, and on the other to pernicious anæmia.

